# Attitudes of fertile and infertile woman towards new reproductive technologies: a case study of Lithuania

**DOI:** 10.1186/1742-4755-11-26

**Published:** 2014-04-01

**Authors:** Aurelija Blaževičienė, Irayda Jakušovaitė, Alina Vaškelytė

**Affiliations:** 1 Nursing and Care Department, Lithuanian University of Health Sciences, Jankaus 2, LT 44307 Kaunas, Lithuania; 2 Social Sciences and Humanity Department, Lithuanian University of Health Sciences, Kaunas, Lithuania

**Keywords:** Fertile women, Infertile women, New reproductive technologies

## Abstract

**Background:**

This article analyzes several key issues in the debate: the acceptability of *in vitro* fertilization; regulation of assisted reproduction and the possibilities of reimbursement for assisted reproduction treatment in Lithuania.

**Method:**

Two groups of respondents participated in the survey: fertile women and women with fertility disorders. 93 completed questionnaires from women with fertility problems and 146 from women with no fertility problems were analysed.

**Results:**

Fertile respondents more frequently perceived the embryo as a human being (Fertile Individuals – 68.5%; Infertile Individuals – 35.5%; p < 0.05) and more frequently maintained that assisted reproduction treatment should be only partly reimbursed (Fertile Individuals – 71.3%; Infertile Individuals – 39.8%; p < 0.05). Respondents with fertility disorders more frequently thought that artificial insemination procedure could also be applied to unmarried couples (Fertile Individuals – 51.4%; Infertile Individuals – 76.3%; p < 0.05), and more frequently agreed that there should be no age limit for artificial insemination procedures (Fertile Individuals – 36.3%; Infertile Individuals – 67.7%; p < 0.05). The majority of respondents in both groups (Fertile Individuals – 77.4%; Infertile Individuals – 82.8%; p < 0.05) believed that donation of reproductive cells should be regulated by law. Fertile respondents more frequently considered that strict legal regulation was necessary in case of the number of transferred embryos (Fertile Individuals – 69.2%; Infertile Individuals – 39.8%; p < 0.05) and freezing of embryos (Fertile Individuals – 69.9%; Infertile Individuals – 57.0%; p < 0.05).

**Conclusion:**

Fertile respondents were statistically more likely to believe that the IVF procedure should be applied only to married couples or women who had a regular partner, the age limit should be defined and the psychological assessment of the couple’s relationship and their readiness for the IVF procedure was necessary. In contrast, infertile couples were statistically more likely than fertile respondents to maintain that the IVF procedure should be fully reimbursed by the state. Fertile respondents were statistically more likely to be categorical with respect to the number of embryos and the freezing of embryos. Meanwhile there is a statistically significant difference in opinions of infertile respondents who were in favour of stricter regulation on donation of reproductive cells.

## Background

According to statistical data, more and more couples around the world including Lithuania complain of fertility disorders or are infertile. In today‘s world every sixth couple, i.e. 10-15%, faces some sort of fertility problems while another 10-25% of women experience secondary infertility, i.e. they cannot conceive following a previous pregnancy. Two million new infertile couples appear annually worldwide [1]. For example, one in six British couples has difficulty in conceiving a baby, and the number of couples seeking medical help to have a family has risen dramatically [2]. At present, in Lithuania there are about fifty thousand infertile couples; two more thousand are added to this number annually. The percentage of men and women that encounter fertility problems is nearly the same. Female infertility constitutes 30-40% of cases, male infertility – 10-30%, infertility due to both partners – 15-30% and unexplained infertility – 5-10% [3].

The solution to this problem is important not only to individual families but also to all countries, including Lithuania, with the decreasing birth rate. In such European countries as Denmark, Finland, Belgium more than 4% of all children are born with the help of assisted reproduction [4] and the best treatment for those with blocked fallopian tubes, or the increasing numbers with unexplained infertility, is *in vitro* fertilization (IVF).

IVF and other forms of assisted reproductive technology have provided society with a wide array of reproductive possibilities that challenge moral and legal conventions regarding the structure of society and the concept of what constitutes the family unit [5]. At the same time, access to health care is increasingly being recognized as a basic human right. If society is required to accept assisted reproduction as a basic right, it has the right to regulate access for its physical, social, and economic well being [5]. The advances of the past decades in the area of reproductive medicine periodically fuel a discussion in the media on the moral boundaries of what is feasible with technology [6]. Researchers have extensive discussions about the moral status of the human embryo [7-10]. Rapid development of biotechnologies leads to new dangers to human life in prenatal phase. Artificial insemination, when embryos are produced *in vitro*, cloning, extracting embryonic stem cedf and experiments with embryos are related to embryonic death. Lithuania has no legal acts regulating the ethical problems associated with infertility treatment and work on such legislation has been in progress for a long time, arousing very intensive emotions in Lithuanian society. Current legislation does not regulate such controversial issues as donation of reproductive cells, freezing, thawing and preservation of embryos, surrogate motherhood. The latest draft of the Law on Assisted Reproduction of the Republic of Lithuania was prepared in 2013; however, due to prolonged debate and dispute it has not yet been adopted. A similar situation may be found in Poland [11].

This article analyzes several key issues in the debate: the acceptability of IVF; regulation of assisted reproduction and the possibilities of reimbursement for assisted reproduction treatment in Lithuania.

## Methods

Two groups of respondents participated in the survey: fertile women and women with fertility disorders. Individuals with fertility problems are united by the Lithuanian Fertility Association which at the time of survey had 122 members who were invited to take part in the survey. The Lithuanian Fertility Association has about 1000 members, however, only those members who were not expecting children and were childless at the time of research were invited to take part in the survey.

In order to ensure full confidentiality of respondents, the Chair of the Fertility Association’s Board personally distributed the questionnaire and collected replies. 93 completed questionnaires were returned and used for further analysis. The response rate is 72.6%.

The group of individuals with no fertility problems was composed of pregnant women who attended regular checkups at one of the Primary Health Care Centres (PHCC) in Vilnius. All pregnant women who during research period attended the PHCCs were invited to participate in the survey.

244 questionnaires were distributed, which is two times more than in the group of women with fertility disorders. 168 questionnaires were returned, 146 of which were suitable for further analysis. The response rate is 59.8%.

For the data analysis the statistical software package "SPSS for Windows 16" was used. An assessment of the correlation between two variables was carried out with the help of statistical reliability tests: Chi square (χ2) criterion and the Z-factor criterion was applied in order to establish the correlation between qualitative variables. The reliability of statistical results/evidence/ was assessed by applying a 0.05 level of significance.

Our participants of our study provided informed consent and permission to conduct study was granted by Bioethical Comity at Lithuanian University of Health Sciences.

## Results

**Figure 1:**
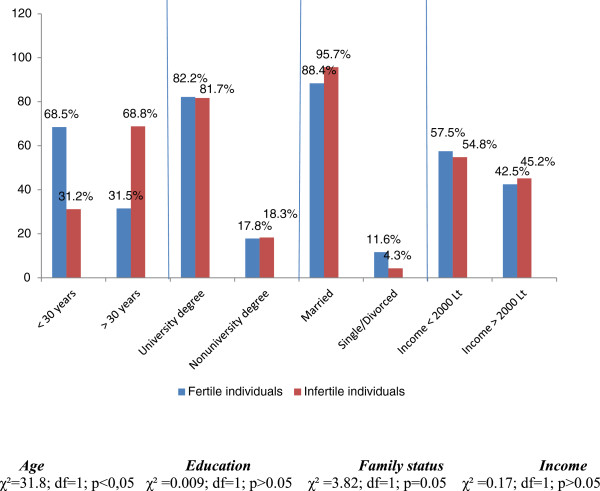
Socio-demographic characteristics of respondents.

The average age of respondents was 29.72 (±0.326) years. The greater part of respondents in both groups had higher education (Fertile Individuals - 82.2%; Infertile Individuals - 81.7%.) and were married (Fertile Individuals – 88.4%; Infertile Individuals – 95.7%) (Figure 1).

A comparison of respondent groups according to socio-demographic characteristics has not revealed any statistically significant differences.

**Figure 2:**
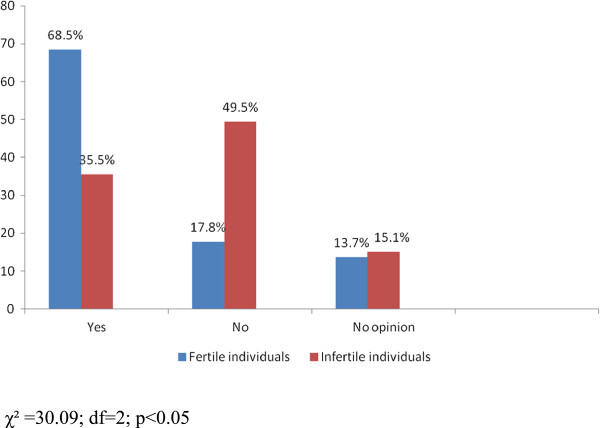
Is the embryo a human being?

This survey analysed the attitudes of fertile respondents and respondents with fertility disorders towards the moral status of the human embryo. The survey data has revealed that there is a statistically significant difference in the attitudes of respondents: fertile respondents more frequently perceived the embryo as a human being (Fertile Individuals – 68.5%; Infertile Individuals – 35.5%; χ^2^ = 30.09; df = 2; p < 0.05) than respondents with fertility problems (Figure 2).

Women who took part in the survey were inclined to freeze the embryos left after the first unsuccessful procedure with the purpose of having less expensive second procedure. A positive response to the question "Do you approve of freezing the embryos in order to preserve them for future use or in case of the failure of the first IVF procedure?" was given by nearly all (93.6%) respondents with fertility disorders and more than a half (63.7%; p < 0.05) of fertile respondents. The most frequently mentioned periods for the preservation of embryos by respondents with fertility disorders was from 5 to 10 years (40.9%) and the currently existing period of preservation for up to 5 years (38.7%).

**Figure 3:**
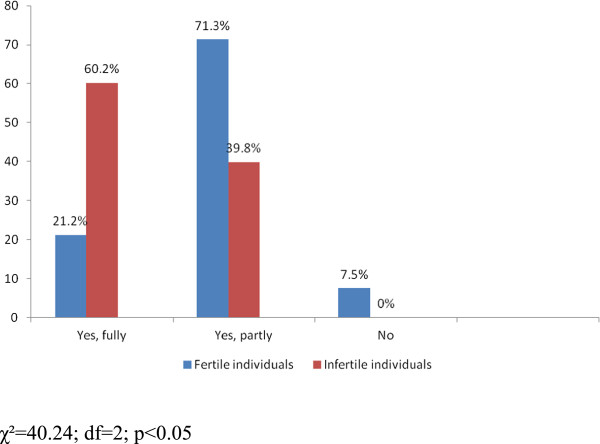
Should assisted reproduction treatment be reimbursed?

Another significant aspect of the analysis of attitudes towards new reproductive technologies is their accessibility which is largely dependent on the price of procedure and the patients’ ability to afford it. Respondents were asked whether the use of new reproductive technologies should be reimbursed. An analysis of data reveals that compared to respondents with fertility disorders fertile respondents more frequently maintained that assisted reproduction treatment should be only partly reimbursed and the difference of opinion was statistically significant (Fertile Individuals – 71.3%; Infertile Individuals – 39.8%; χ^2^ = 40.24; df = 2; p < 0.05) (Figure 3).

Respondents were given the set of questions aiming to establish their opinion on the ethical issues of artificial insemination as well as its acceptability to respondents. Based on the survey data it may be argued that respondents with fertility disorders more frequently than fertile respondents (Fertile Individuals – 51.4%; Infertile Individuals – 76.3%; χ^2^ = 15.54; p < 0.05) thought that artificial insemination procedure could also be applied to unmarried couples. Also respondents of this group (Fertile Individuals – 42.5%; Infertile Individuals – 82.8%; χ^2^ = 40.37; p < 0.05) did not think that the psychological assessment of the couple’s relationship was necessary before artificial insemination procedure and more frequently agreed that there should be no age limit for artificial insemination procedures (Fertile Individuals – 36.3%; Infertile Individuals – 67.7%; χ^2^ = 22.47; p < 0.05). Respondents in both groups (Fertile Individuals – 57.5%; Infertile Individuals – 65.6%; χ^2^ = 1.71; p < 0.05) unanimously agreed that single women who did not have a regular partner should be able to have artificial insemination procedure without any limitations (Table 1).

**Table 1:** Ethical questions of assisted reproduction treatment and its acceptability to respondents

**Statement**		**Yes (%)**	**No (%)**	**No opinion (%)**	**χ**^**2**^**, p**
IVF should be applied only to married couples	Fertile	39.7	51.4	8.9	p < 0.05
	Infertile	17.2	76.3	6.5	χ^2^ = 15.54
Psychological assessment of the couple’s relationship should be necessary before IVF procedure	Fertile	48.6	42.5	8.9	p < 0.05
	Infertile	10.8	82.8	6.5	χ^2^ = 40.37
Age limit should be imposed for those seeking IVF procedures	Fertile	55.5	36.3	8.2	p < 0.05
	Infertile	28.0	67.7	4.3	χ^2^ = 22.47
Single women without a regular partner but willing to conceive have a right to take advantage of the sperm bank and assisted reproduction treatment	Fertile	57.5	32.2	10.3	p < 0.05
	Infertile	65.6	24.7	9.7	χ^2^ = 1.71

An analysis of respondents’ attitudes towards legal provisions on new reproductive technologies has shown that the majority of respondents in both groups (Fertile Individuals – 77.4%; Infertile Individuals – 82.8%; χ^2^ = 1.02; df = 1; p < 0.05) believed that donation of reproductive cells should be regulated by law. There was a statistically significant difference in opinions expressed by fertile respondents who more frequently considered that strict legal regulation was necessary in case of the number of transferred embryos (Fertile Individuals – 69.2%; Infertile Individuals – 39.8%; χ^2^ = 20.12; df = 1; p < 0.05) and freezing of embryos (Fertile Individuals – 69.9%; Infertile Individuals – 57.0%; χ^2^ = 4.14; df = 1; p < 0.05) (Table 2).

**Table 2:** Respondents’ opinion as to what matter should be regulated by the law on assisted reproduction

**Regulation necessary**	**Fertile individuals (%)**	**Infertile individuals (%)**	**χ**^**2**^**, df, p**
The number of transferred embryos	69.2	39.8	χ^2^ = 20.12
			df = 1; p < 0.05
Freezing of embryos	69.9	57.0	χ^2^ = 4.14
			df = 1; p < 0.05
Donation of reproductive cells	77.4	82.8	χ^2^ = 1.02
			df = 1; p < 0.05

## Discussion

Attitudes of fertile and infertile women towards new reproductive technologies depend on the answer to the question whether an embryo is a human being or merely a human entity.

For some people, particularly for those adhering to religious creeds, "human embryos are not more biological tissues or clusters of cells; they are the tiniest of human beings, thus we have a moral responsibility not to deliberately harm them" (Center for Bioethics and Human Dignity, 1999). For others - among them the overwhelming majority of the biologist community - an early embryo, prior to development of the primitive streak or implantation, is a ball of cells, not a human entity, and the embryo research oriented to finding cures for a number of major diseases is morally legitimate. In modern societies, beliefs about the *moral status of the embryo* have been shaped by several cultural traditions, in particular by scientific advances in biology and medicine and, more importantly, by religion [12-16]. Data of this research reveals that more than a half of fertile respondents and one third of respondents with fertility disorders believed an embryo is a fully formed human being. Based on this, it may be presumed that respondents are more likely to support the Catholic point of view towards the moral status of an embryo.

The objective of a health system is to deliver health care to all those in need. However, in some Western countries, the limitless demand for health care can often not be met due to the scarcity of resources to service it. Governments in some countries that reimburse assisted reproductive technology (ART) treatment, such as Austria, France and the United Kingdom, impose age criteria. Israel and Ireland is currently debating this issue [17-21].

Equity in health care means equal access to basic health care without excessive burdens. Given the importance of health for the general well-being of a person, every person, regardless of his or her income or financial means, should have access to a decent minimum of health care. The first question is whether assistance to reproduction should be included in basic health care and thus can be eligible for reimbursement [22]. The 2007 European IVF Monitoring (EIM) annual report indicates that the artificial insemination procedure is reimbursed to a different degree in 23 countries [23]. Research data has shown that lower reimbursement was associated with fewer cycles per 100,000 population (slope = 7.2, p = 0.004). There was no association between reimbursement level and the percent of IVF patients who were less than 34 years old (p = 0.51) or >40 years old (p = 0.89).

Consumption rates of assisted reproductive technologies in Israel is internationally unprecedented, a phenomenon that has been the subject growing anthropological and sociological attention. Case study analysis of public dispute that took place in 2003 - 2004 over the extent of public funding for fertility treatment and has been in three sites: the parliament, the media and community [24-26]. The main question on the answer to which the government’s and the society’s attitude on the reimbursement of procedure is dependent is whether infertility is a social or medical problem. Data of our survey demonstrates that the majority of respondents with fertility disorders consider infertility to be medical problem and approve of the reimbursement of assisted reproduction treatment. In contrast, fertile respondents only partly agreed that this procedure should be reimbursed from the state budget. A study carried out by researchers from Australia and California where couples from the United States, Canada, United Kingdom, Scandinavia, Japan, and Australia took part, has revealed that the cost of ART treatment did not exceed 0.25% of total healthcare expenditure in any country. Researchers concluded that assisted reproductive technology is expensive from a patient perspective but not from a societal perspective [27,28].

One of the basic human rights is that of a woman to decide when and how to conceive. Under the European Convention, a single woman or even a lesbian couple is entitled to have children, even though these children may have no legal father [21,28]. Unprecedented situation is in Israel. Explanation for the singular extent of ART’s use in Israel tend to pre-assume and conceptually prioritize the symbolic and political power of pro-natalist discourse Jewish religious values and demographic interests of the Jewish state [13].

The question "Who shall be eligible for assisted reproductive technologies?" signals the onerous has been discussion about whether it is ethical to allow single women, lesbian or homosexual couples access to ART. Many believe that this is morally wrong, arguing that it is preferable for a child to be raised within a stable, heterosexual relationship [29]. Most professional bodies and legislation in the various countries of Europe have recommended that ART should be restricted to heterosexual couples, legally married, or at least living in a stable relationship. Nearly half of fertile respondents of this survey had quite liberal opinions on age limit, marital status and compulsory psychological consultations prior to the use of assisted reproductive technologies. Although data of our survey has revealed that more than two thirds of infertile couples believed there was no need for the psychological assessment, however, studies carried out in other countries have shown the opposite results.

Kainz, Wischmann conducted studies aiming to assess whether it was necessary for infertile couples to have a consultation by a psychologist or a mental health professional prior to the IVF. Study results have shown that psychologist, or other mental health professional on the health care team, is essential to treatment of the biopsychosocial nature of infertility [29-31].

Based on the results of the Women and Health Survey and the Psychosocial Infertility Interview Study which were carried out by the Danish researchers it may be maintained that a psychological consultation is essential to infertile couples, moreover, it is necessary to develop various psychological interventions that would help infertile couples [32]. Another important aspect in discussion on new reproductive technologies is the legal framework. In the study conducted by the US researchers already 20 years ago the majority of respondents (64%) maintained that live embryo freezing should be permitted by law. Overall, 72% of the respondents thought that ART should be regulated [32].

Results of this survey have revealed that fertile women had stronger views and wished that the number of transferred embryos as well donation of reproductive cells should be explicitly regulated by law. In contrast, respondents with fertility disorders had more liberal views on the number of transferred and frozen embryos but were in favour of strict regulation of donation.

### Strengths and weaknesses of the study

The strength of this study is that it is one of the few studies on issues of infertility conducted in Lithuania. As it has already been noted, so far in Lithuania no law on reproductive health has been adopted, which would possibly allow for society to become more involved into a debate on infertility.

The weakness of the study is that the survey sample is limited in number since the majority of infertile couples would like to remain anonymous and preserve their confidentiality. Another limitation of this study is that only families that are members of the Lithuanian Fertility Association participated in the survey. Furthermore, the survey was dominated by female respondents while it would be beneficial to find out the opinions and suggested solutions provided by male respondents.

### Clinical and policymakers implications

Infertility and ARTs pose challenges not just for fertility specialists, but also for general practitioners, gynecologists and others providing care for people with fertility concerns.

Women initiate most fertility inquiries, both because they see their physicians more frequently and because social norms assign them greater reproductive responsibility. Their male partners should never be ignored, however, either medically or emotionally.

According to opinions of respondents, politicians should speed up the adoption of the law on reproductive health which would define the reimbursement procedure, the number of embryos to be frozen and the principles of donation of reproductive cells.

## Conclusions

In summary, it may be maintained that fertile respondents were statistically more likely to believe that the IVF procedure should be applied only to married couples or women who had a regular partner, the age limit should be defined and the psychological assessment of the couple’s relationship and their readiness for the IVF procedure was necessary.

In contrast, infertile couples were statistically more likely than fertile respondents to maintain that the IVF procedure should be fully reimbursed by the state. While expressing opinions on legal and ethical regulation of IVF, fertile respondents were statistically more likely to be categorical with respect to the number of embryos and the freezing of embryos. Meanwhile there is a statistically significant difference in opinions of infertile respondents who were in favour of stricter regulation on donation of reproductive cells.

Data of this research very well reflects the current situation in Lithuania: one part of society supports the more liberal version of the law on reproductive health, while the other part of society is in favour of its more conservative version. Unfortunately, the national systemic approach towards solving problems of assisted reproduction is lacking in Lithuania.

## Competing interests

The authors declare that they have no competing interests.

## Authors’ contributions

AB and IJ conceived the study. AV analyzed the data. AB, IJ and AV drafted the manuscript and reviewed the article. All authors extensively reviewed the article and read and approved the final manuscript.
